# Risk Factors of Caregiver Burden in Patients With Cognitive Dysfunction: A Scoping Review

**DOI:** 10.1002/brb3.71191

**Published:** 2026-01-07

**Authors:** Liu Jinheng, Zhao Jingyi, Cui Shaomei, Ye Danjuan, Wang Liansheng, Chen Lixia

**Affiliations:** ^1^ Department of Nursing, the Fourth Affiliated Hospital of School of Medicine, and International School of Medicine, International Institutes of Medicine Zhejiang University Yiwu China

**Keywords:** caregiver burden, cognitive dysfunction, risk factors, scoping review

## Abstract

**Introduction:**

To synthesize the risk factors of caregiver burden in patients with cognitive dysfunction and provide insights for clinical nursing practice and research.

**Methods:**

A scoping review was conducted following the five‐stage methodological framework proposed by Arksey and O'Malley. A systematic search was performed using a combination of subject terms and free terms across six databases: China National Knowledge Infrastructure, Wanfang Database, PubMed, Embase, Cochrane Library, and Web of Science Core Collection. The search period covered from the establishment of each database to July 22, 2025, with grey literature excluded. Two authors independently screened the literature based on predefined inclusion and exclusion criteria, and discrepancies were resolved by team consensus. Data from included studies were extracted into tables, and results were collated through descriptive overview and thematic synthesis.

**Results:**

A total of 3313 records were retrieved, and 19 studies were finally included. Included studies covered regions such as China, the United States, Japan, Australia, and several European countries and involved 5233 patients with cognitive dysfunction and 5586 caregivers. Caregiver burden was influenced by three categories of risk factors: patient factors, caregiver factors, and environmental‐social factors. The most commonly used tools to measure caregiver burden were the Zarit Burden Interview and Caregiver Burden Inventory.

**Conclusions:**

Caregiver burden in patients with cognitive dysfunction is the result of multifactorial interactions, with neuropsychiatric symptoms and behaviors of patients as core risk factors and patient functional dependence and caregiver female gender as driving risk factors. These factors increase caregiver burden by enhancing care intensity, amplifying psychological pressure, and reducing coping resources. Future research should develop comprehensive intervention models based on cross‐cultural comparisons and longitudinal designs to reduce caregiver burden.

## Graphical abstract

This scoping review identifies neuropsychiatric symptoms and behaviors as the core risk factor for caregiver burden in cognitive dysfunction, with functional dependence and female caregivers as key drivers. Findings underscore the need for targeted, multi‐domain interventions to alleviate burden across diverse caregiving contexts.

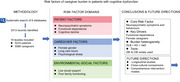

## 1 Introduction

The global prevalence of mild cognitive impairment (MCI) in the older population aged 65 years and over is estimated to be around 10–20%, rising significantly with age, with around 10–15% of people with MCI progressing to dementia each year (Petersen et al. 2014). The number of people with dementia continues to increase as the global population grows and population aging accelerates, with an estimated 57.4 million people with dementia in 2019 globally, and this number is projected to reach 83.2 million in 2030, increasing to 153 million in 2050 (Nichols et al. 2022). Alzheimer's disease (AD) and other dementias were the fourteenth leading cause of Disability‐Adjusted Life Years (DALYs) globally in 2022, and are projected to rise to tenth place by 2050 (GBD 2021 Forecasting Collaborators 2024).

Cognitive dysfunction is defined as reduced or impaired mental and/or intellectual functioning (Huang et al. 2025). It encompasses seven core cognitive domains: general intelligence, executive functioning, memory, attention, psychomotor speed, visuospatial functioning, and language. Cognitive dysfunction has been observed in a variety of neurodegenerative disorders, such as AD, frontotemporal dementia (FTD), dementia with Lewy bodies (DLB), and Parkinson's disease (PD) (Zhang et al. 2022). In this review, “cognitive dysfunction” is used as the primary overarching term. Where the term “cognitive impairment” appears, it reflects the specific language of cited sources or describes a broader context. There is no consensus on the diagnostic criteria for cognitive dysfunction: some studies define it using age‐related cognitive decline (Wang et al. 2024), while others use the Montreal Cognitive Assessment (MoCA) for detection (Islam et al. 2023). According to the fifth edition of the Diagnostic and Statistical Manual of Mental Disorders (DSM‐5), cognitive impairment can be differentiated into mild cognitive impairment (MCI, mild‐to‐moderate) and dementia (severe) based on history, examination, and appropriate objective assessments (Hugo and Ganguli 2014). AD is the leading cause of dementia (Davis et al. 2018).

As cognitive dysfunction progresses from mild to severe dementia, caregiving responsibilities increase substantially. These responsibilities often include assisting with daily living activities, managing neuropsychiatric symptoms, and providing 24‐h supervision, which collectively contribute to significant physical, emotional, and financial burdens for caregivers. Cognitive dysfunction in people with dementia is often accompanied by changes in mood, emotional control, behavior, or motivation. Dementia has physical, psychological, social, and economic impacts, not only for people with dementia but also for their caregivers, families, and society at large (World Health Organization 2023). It has been noted that caregivers bear a significant caregiver burden in providing care for people with mental health conditions (Gharavi et al. 2018), and that the caregiver burden for people with cognitive dysfunction is significantly higher than for other chronic disease groups (Demirbas et al. 2023).

Caregiver burden refers to the multidimensional response of caregivers to the physical, psychological, emotional, social, and financial stressors associated with the care experience (Nemcikova et al. 2023). It has negative outcomes for both caregivers and the care recipients. Caregivers may experience stress, strain, depression, and reduced subjective well‐being (Liu et al. 2020; Reuben et al. 2022; Stall et al. 2019), while care recipients may face abuse, institutionalization, condition deterioration, and reduced quality of life (Stall et al. 2019).

Despite the well‐documented significance of this issue, existing reviews on caregiver burden in cognitive dysfunction have critical limitations. Traditional reviews have mostly focused on a single type of cognitive dysfunction (Connors et al., 2019; Goto et al., 2023; Paradise et al., 2015; Rebolo et al., 2025; Springate and Tremont, 2013), and failed to encompass all types of cognitive impairment. The compositional dimensions of caregiver burden for people with cognitive dysfunction and the comparison of longitudinal studies (Liu et al. 2022) still lack systematic integration. Furthermore, the publication of a growing number of new studies on this topic underscores the need for a timely scoping review update.

To address these unresolved gaps, we conducted a scoping review. Scoping reviews help to organize and summarize the scope and characteristics of the existing literature on a particular topic, as well as identify knowledge gaps in research on a particular topic to assist in planning future research (Tricco et al. 2016). Unlike systematic reviews, scoping reviews do not focus on the quality of the included literature but support the identification of the wider range of relevant studies (Tricco et al. 2018). To this end, we conducted a scoping review to comprehensively gather existing research evidence on caregiver burden for people with cognitive dysfunction, synthesize and systematically describe the risk factors of caregiver burden across all types of cognitive dysfunction, and identify research gaps to inform targeted interventions and policy development.

## 2 Methods

The aim of this study was to scope the literature to identify current risk factors of caregiver burden for people with cognitive dysfunction. The study followed the five stages outlined in Arksey and O'Malley's methodological framework (Arksey et al., 2005)

### 2.1 Identification of Research Questions

The research questions of interest for this scoping review were as follows: (1) What are the risk factors that increase the likelihood or severity of caregiver burden for people with cognitive dysfunction? (2) What are the existing tools for measuring caregiver burden for people with cognitive dysfunction? (3) What are the research gaps in the study of risk factors for caregiver burden in people with cognitive dysfunction to guide future research?

### 2.2 Study Selection

Literature inclusion criteria: (1) The study population was patients with cognitive dysfunction and their caregivers, and the types of patients with cognitive dysfunction included inpatients, community patients, and nursing home patients; (2) The study consisted of original literature on the caregiver burden of cognitive dysfunction patients; (3) The types of studies included cross‐sectional, longitudinal, cohort, and case‐control studies; (4) The sources of information were restricted to peer‐reviewed articles published in academic journals.

Exclusion criteria were (1) duplicates; (2) articles with unavailable full text or incomplete data; (3) non‐English and Chinese literature; and (4) reviews, conference abstracts, dissertations, editorials, letters, and books.

The literature screening process included the use of automated methods to identify duplicate articles. In addition, two authors independently assessed the titles, abstracts, and full texts of the identified articles. A third reviewer was consulted if consensus could not be reached. As is typical for scoping reviews, no formal quality assessment of included studies was conducted, as the goal was to map the breadth of literature on risk factors rather than evaluate methodological rigor (Tricco et al. 2018).

### 2.3 Identification of Relevant Studies

A comprehensive search was conducted in six databases using a combination of subject terms plus free terms, that is,China National Knowledge Infrastructure (CNKI), Wanfang Database, PubMed, Embase, Cochrane Library, and Web of Science Core Collection. The search covered the period from the inception of each database to July 22, 2025. No language filters were applied during the database search; the limitation to English and Chinese literature was implemented during the record screening process. The search strategy was designed to be exhaustive and encompass the broad terminology used in the literature. In PubMed's MeSH database, “Cognitive Dysfunction” is the designated Medical Subject Heading (MeSH) for the concept that encompasses “Cognitive Impairment” and related terms. Therefore, our strategy intentionally incorporated both the precise MeSH term “Cognitive Dysfunction” and the more widely used textword “cognitive impairment” (along with its variants) to synergistically leverage the high precision of controlled vocabulary and the broad recall of free‐text searching. This approach ensured comprehensive coverage of the relevant evidence. The comprehensive search strategy was developed by systematically trained nursing postgraduates and further refined through team consensus. However, grey literature was excluded from the search. This decision was made a priori due to practical constraints, including resource and time limitations, and to maintain a focused scope on peer‐reviewed, primary research evidence that has undergone formal editorial scrutiny. The results of the literature search were imported into EndNote x9.3.3. English search terms included “Cognitive Dysfunction*/Cognitive Disorder*/Cognitive Impairment*/Mild Cognitive Impairment*/Cognitive Decline*/Mental Deterioration*/cognitive defect*/cognitive complaints/cognitive deficiency/cognitive deficit/cognitive difficulties/cognitive disability/cognitive disturbance/cognitive dysfunction/response interference/cognitive problems/,”“Caregiver Burden/Care Giving Burden/Caregiver Burden/Caregiver Stress/Caregiver Stresses/Caregiver Exhaustion/Caregiving Burnout*/Care Burden/Caregiving Stress/Caregiving Stresses/Caregiver Burnout/Caregiver overload/Caregiver strain/Caregiver's burden/Caregiver's strain/ caregiver's stress/caregivers burden/caregivers overload/caregivers strain/caregivers stress/caregiver burden.” Chinese search terms include “认知功能障碍/轻度认知功能障碍/术后认知功能障碍/认知障碍/阿尔兹海默病/阿尔兹海默症/轻度认知障碍/'MCI/老年痴呆/痴呆“”照顾者负担/照顾负担/照顾压力/照顾者压力.” The search strategy for PubMed is provided in Table 1 as an example.

**TABLE 1:** Search strategy (PubMed as an example).

Step	Search term	Field/Type
#1	“Cognitive Dysfunction”	[MeSH Terms]
#2	“Cognitive dysfunction*” OR “cognitive disorder*” OR “cognitive impairment*” OR “mild cognitive impairment*” OR “cognitive decline*” OR “mental deterioration*” OR “cognitive defect*” OR “cognitive complaints” OR “cognitive deficiency” OR “cognitive deficit” OR “cognitive difficulties” OR “cognitive disability” OR “cognitive disturbance” OR “response interference” OR “cognitive problems”	[Title/Abstract]
#3	#1 OR #2	—
#4	“Caregiver Burden”	[MeSH Terms]
#5	“Care Giving Burden” OR “caregiver burden*” OR “Caregiver Stress” OR “Caregiver Stresses” OR “Caregiver Exhaustion” OR “Caregiving burnout*” OR “Care Burden” OR “Caregiving Stress” OR “Caregiving Stresses” OR “Caregiver Burnout” OR “caregiver overload” OR “caregiver strain” OR “caregiver's burden” OR “caregiver's strain” OR “caregiver's stress” OR “caregivers burden” OR “caregivers overload” OR “caregivers strain” OR “caregivers stress”	[Title/Abstract]
#6	#4 OR #5	—
#7	#3 AND #6	—

^a^ *Note*: The search strategy was adapted for each database based on its specific syntax (e.g., Embase uses “EMTREE Terms” instead of MeSH Terms; CNKI uses Chinese subject headings). No publication date filters were applied; language restrictions (English/Chinese) were applied during the screening stage.

### 2.4 Data Extraction

A standardized data extraction form was developed a priori and piloted on two included studies to ensure consistency. The following data were extracted from each included study by two independent authors: author(s), publication year, country/region, study design, sample size (number of patients and caregivers), type of cognitive dysfunction, caregiver‐patient relationship, caregiver burden measurement tools, and all reported risk factors (with direction of effect and supporting statistical measures where available). Any discrepancies in extraction were resolved through discussion and consensus between the two reviewers, with the option to consult a third reviewer if necessary.

### 2.5 Collating, Summarizing and Reporting Results

Extracted data were synthesized using a convergent two‐step approach. First, Descriptive Synthesis: Study characteristics (e.g., design, location) were summarized narratively and using frequency counts (n, %) to profile the evidence base. The prevalence of each reported risk factor was quantified by counting the number of supporting studies. Second, Thematic Synthesis: risk factors were systematically categorized into an analytical framework comprising three overarching themes: patient‐related, caregiver‐related, and environmental‐social factors. Within these themes, specific sub‐themes were derived inductively from the data. The thematic synthesis was conducted independently by two reviewers to minimize bias and enhance the reliability of the findings. Any discrepancies in thematic categorization were resolved through structured consensus discussions during regular team meetings. A third senior reviewer was available to arbitrate in the rare event that a consensus could not be reached between the initial two reviewers. The synthesized results are reported in accordance with the PRISMA‐ScR guidelines. They are presented through integrated narrative summaries that are substantiated by tables and figures.

## 3 Results

### 3.1 Search Results

**FIGURE 1:**
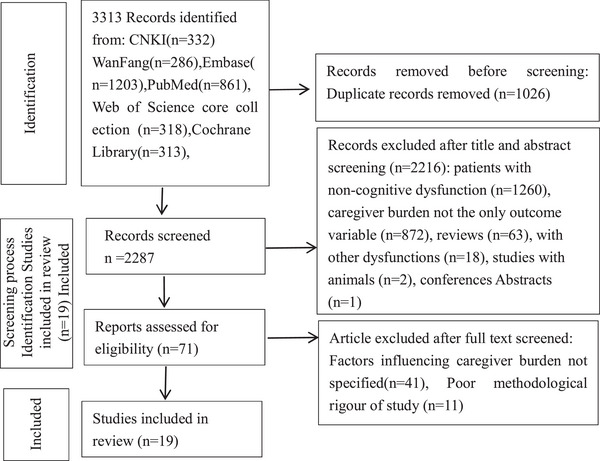
Identification of studies via databases and registers.

A total of 3313 records were obtained through a comprehensive search of the above databases. After careful screening based on predetermined inclusion and exclusion criteria, 19 relevant pieces of literature were finally included in this study. The literature screening process is shown in Figure 1.

### 3.2 Essential Features of the Included Studies

A summary of the essential features of the 19 included studies is presented in Table 2.

**TABLE 2:** Main characteristics of included studies (*n* = 19).

Author(s) (Year)	Geographic distribution	Study design	Sample size	Cognitive dysfunction type	Caregiver burden Measurement Tool(s)	Study aim(s)
Allegri et al. (2006)	Argentina	Cross‐Sectional study	82 patient/caregiver dyad	AD	ZBI	Assessing predictors of caregiver burden in Argentinean patients with Alzheimer's disease
Germain et al. (2009)	Multi‐country European	Longitudinal study	1091 patient/caregiver dyad	AD	ZBI	Identifying key predictors of caregiver burden in Alzheimer's disease and exploring the impact of cognitive impairment on it
Agüera‐Ortiz et al. (2010)	Spain	Multicentre observational study	1235 patient/caregiver dyad	AD	ZCBS	To analyze the relationship between clinical progression of AD and caregiver burden
Chen et al. (2011)	China	Cross‐Sectional study	54 caregivers	AD	CBI	Investigating the status of caregiver burden and influencing factors of dementia patients
Hayashi et al. (2013)	Japan	Cross‐Sectional study	104 patient/caregiver dyad	MCI	sZBI	To assess the current status of caregiver burden among patients with MCI, clarify the association between MCI patient characteristics and caregiver burden
Shankar et al. (2014)	USA	Cross‐Sectional study	495 patient/caregiver dyad	Dementia, delirium or both	CBI	Factors associated with the burden experienced by older people with cognitive impairment at the time of hospitalization and their caregivers
D'Onofrio et al. (2015)	Italy	Cross‐Sectional study	506 patient/caregiver dyad	AD, VaD	CBI	Examining differences in caregiver burden for people with AD and VaD to improve care advice and management programs
Wu et al. (2015)	China	Cross‐Sectional study	101 patient/caregiver dyad	AD	ZBI	Exploring the burden and impact of dementia events on primary caregivers and the patient's family
Yu et al. (2015)	China	Cross‐Sectional study	168 patient/caregiver dyad	AD	CBI	Assessing the caregiver burden of caregiving community‐dwelling patients with mild Alzheimer's disease and exploring the direct and indirect effects of patient or caregiver factors on it
Wang et al. (2016)	China	Cross‐Sectional study	152 caregiver	AD	CBI	Exploring the burden on family caregivers of patients with dementia in the community and its influencing factors
Xu et al. (2016)	China	Cross‐Sectional study	212 patient/caregiver dyad	AD	ZBI, CSI	Exploring the current status and factors influencing family caregiver burden and stress in people with dementia
van der Lee et al. (2017)	The Netherlands	Longitudinal study	148 caregiver	AD, Amnesia, or other type	VAS, NPI	Explore which patient and caregiver characteristics determine different types of caregiver burden over time
Connors et al. (2019)	Australia	Longitudinal study	185 patient/caregiver dyad	MCI	ZBI	Examining the prevalence and predictors of caregiver burden in people with MCI
He et al. (2020)	China	Cross‐Sectional study	97 patient/caregiver dyad	AD	CBI	To understand the current situation of family caregiver burden, factors influencing it, and the demand and utilization of health services for people with Alzheimer's disease
Hergert et al. (2021)	USA	Cross‐Sectional study	50 patient/caregiver dyad	HD	CAS	Exploring the impact of disease characteristics of HD on caregiver burden, predictors of caregiver burden in Huntington's disease patients
Iravani et al. (2022)	Iran	Cross‐S study	85 patient/caregiver dyad	AD	CBI	Assessing Neuropsychiatric Symptoms in Patients with AD and Their Relationship to Caregiver Burden
Kang et al. (2022)	Australia	Cross‐Sectional study	71 patient/caregiver dyad	YOD	ZBI	To assess the level of burden and psychological distress among caregivers of people with YOD in a state tertiary service in Australia and to identify factors that predict the level of caregiver burden in the YOD group.
Yuuki et al. (2023)	Japan	Cross‐Sectional study	593 patients (93 DLB,500 AD) + caregivers	DLB, AD	J‐ZBI	To compare the level of caregiver burden in patients with DLB and AD and to explore the factors affecting caregiver burden in the two groups separately
Yu et al. (2024)	China	Cross‐Sectional study	157 patient/caregiver dyad	AD	ZBI	To study the burden of caregivers of elderly people with dementia in nursing facilities and to analyze the influencing factors

**Abbreviations**: CAS, Caregiving Appraisal scale; CBI, Caregiver burden inventory; CSI, Caregiver stress inventory; DLB, Dementia with lewy bodies; HD, Huntington's disease; J‐ZBI, Japanese version of the Zarit caregiver burden interview; NPI, Neuropsychiatric inventory; sZBI, Short Japanese version of Zarit burden interview; VaD, Vascular dementia; VAS, Visual analogue scale; YOD, Young‐onset dementia; ZBI, Zarit's burden interview; ZCBS, Zarit Caregiver Burden Scale.

#### 3.2.1 Study Characteristics

A total of 19 studies on the risk factors of caregiver burden in patients with cognitive dysfunction were published between 2006 and 2024. Among them, 3 studies were published in 2015, 2 each in 2016 and 2022, and 1 each in 2006, 2009, 2010, 2011, 2013, 2014, 2017, 2019, 2020, 2021, 2023, and 2024.

#### 3.2.2 Geographic Distribution

Geographically, the studies were conducted in China (*n* = 7, 36.8%), United States, Japan, Australia (*n* = 2 each, 10.5% each), the Netherlands, Spain, Italy, Iran, Argentina (*n* = 1 each, 5.3% each), and a multi‐country European study (*n* = 1, 5.3%) involving 12 countries (Belgium, the United Kingdom, France, Denmark, Germany, Greece, Italy, Romania, Spain, Sweden, Switzerland, and the Netherlands).

#### 3.2.3 Sample and Study Design

The 19 studies included 5233 patients with cognitive dysfunction and 5586 caregivers. 16 studies collected data from patient‐caregiver pairs, and 3 studies collected data only from caregivers. Study designs included: cross‐sectional (*n* = 15, 78.9%), longitudinal (*n* = 3, 15.8%). Mixed cross‐sectional/prospective observational (*n* = 1, 5.3%).

#### 3.2.4 Measurement Tools

The most commonly used tools to measure caregiver burden were the Zarit Burden Interview (ZBI) and its derivatives (J‐ZBI, sZBI): *n* = 9 (47.4%), the Caregiver Burden Inventory (CBI): *n* = 7 (36.8%), and other tools (ZCBS, VAS+NPI, CAS): *n* = 3 (15.8%).

### 3.3 Risk Factors Influencing Caregiver Burden for People With Cognitive Dysfunction

The influencing factors of caregiver burden were categorized into three groups: patient factors, caregiver factors, and environmental‐social factors. A detailed synthesis is provided in Table 3, and a summary of key findings is presented below.

**TABLE 3:** Summary of risk factors influencing caregiver burden.

Category	Subcategory	Specific risk factor	Number of supporting studies (*n*)	Study design (Cross‐sectional/Longitudinal)	Mechanism of Action
Patient factors	Neuropsychiatric symptoms and behaviors	Delirium, anxiety, irritability, apathy, restlessness, hallucinations, delusions, depression, level of neuropsychiatric symptoms	14	11/3	Disrupts daily care routines; requires additional time/energy for symptom management; elevates caregiver psychological stress
		Behavioral disinhibition, abnormal motor behaviors, behavioral disorders, severe behavioral symptoms, number of behavioral problems	6	5/1	Compromises care predictability; increases physical and emotional demands on caregivers
	Memory functioning	Severe memory impairment	1	1/0	Necessitates repeated reminders and assistance with daily activities
	Functional ability	Impaired basic activities of daily living	4	3/1	Raises physical care workload; reduces patient independence
		Impaired instrumental activities of daily living	3	2/1	Requires caregiver substitution for complex daily tasks
	Cognitive function and disease	Longer duration of illness	2	1/1	Leads to cumulative care demands and progressive patient functional decline
		Severe dementia	1	1/0	Exacerbates all care needs
		Severe cognitive decline	6	4/2	Reduces patient communication and self‐care capacity; increases caregiver decision‐making burden
	Demographic and sociological characteristics	Male gender	2	1/1	May be associated with higher likelihood of aggressive behaviors or lower adherence to care routines
		Insufficient sleep time (<6 h/d)	1	1/0	Causes nighttime care disruptions and caregiver sleep deprivation
		Higher educational level	1	1/0	May reflect unmet expectations for patient functional status or care complexity
Caregiver factors	Socio‐demographic characteristics	Female gender	6	5/1	Greater emotional investment in care; disproportionate allocation of care tasks; higher emotional vulnerability
		Younger age	2	2/0	Role conflict (balancing care with work/family responsibilities); limited caregiving experience
		Spousal relationship with patient	1	1/0	Closer emotional bond leading to greater grief/distress; lifelong care expectations
		Parent‐child relationship with patient	1	1/0	Cultural or filial responsibility pressures; emotional attachment to parent
		Kinship with patient (other relatives)	1	1/0	Potential lack of prior caregiving experience; ambiguous care role boundaries
		Insufficient funds at the end of the month	1	1/0	Financial stress from out‐of‐pocket care expenses
		Heavy economic burden	3	3/0	Compromises access to supportive services; creates financial anxiety
		Married	1	1/0	Balancing spousal care with family responsibilities; limited personal time
		Employed	1	1/0	Work‐care conflict; time constraints; fatigue from dual roles
		Unemployed or housewife	1	1/0	Financial dependence; lack of social support outside caregiving; role isolation
		Poor health status	3	2/1	Limited physical capacity to meet care demands; caregiver health deterioration
		Low health‐related quality of life	1	1/0	Pre‐existing physical/mental health issues exacerbated by caregiving
		Mental health problems	1	1/0	Reduced coping capacity; emotional exhaustion from dual burden
		Low educational level	3	3/0	Limited access to caregiving resources/information; reduced problem‐solving skills
		Higher educational level	1	1/0	Internalized responsibility; higher expectations for care quality; “high expectation pressure” in collectivist cultures
		High affinity personality	1	1/0	Over‐empathy leading to emotional distress; difficulty setting care boundaries
	Psychological factors	Depressive symptoms	1	1/0	Diminished motivation and energy for caregiving; negative emotional spillover
		Excessive emotional support‐seeking behaviors	1	1/0	Strains social support networks; creates additional emotional labor for others
		Heavy psychological stress	1	1/0	Chronic stress response; impairs decision‐making and care quality
		Low symptom management self‐efficacy	1	1/0	Lack of confidence in handling patient symptoms; heightened anxiety during care crises
	Care‐related factors	Longer care duration	6	4/2	Prolonged physical and mental exhaustion; cumulative stress
		Care duration >8 h/d or 61 h/week	1	1/0	Intensive, unrelenting care demands; no adequate rest or recovery time
		Living with the patient	1	1/0	24/7 care availability expectations; blurred work‐life boundaries
		Low sense of competence	1	1/0	Self‐doubt about caregiving effectiveness; feelings of inadequacy
		Lack of professional training	1	1/0	Insufficient skills to manage complex symptoms; higher risk of care errors
		Undertaking additional housework	1	1/0	Dual workload (caregiving + household chores); no division of labor
		Low number of shared caregivers	2	2/0	Concentration of care tasks on single caregiver; no respite opportunities
Environmental‐social factors	Social support	Low social support	1	1/0	Lack of practical/emotional assistance; isolation in caregiving role
	Family functioning	Poor family functioning	1	1/0	Reduced mutual assistance; caregiver role strain; emotional discord in family

#### 3.3.1 Patient Factors

These factors are primarily derived from 14 cross‐sectional studies and 3 longitudinal studies, with many studies reporting on multiple factors. Neuropsychiatric symptoms and behaviors were identified as the most consistent core risk factors.

- Neuropsychiatric symptoms and behaviors: 14 studies confirmed that symptoms such as hallucinations, delusions, anxiety, and abnormal motor behaviors increase caregiver burden (Allegri et al. 2006; Connors et al., 2019; D'Onofrio et al. 2015; Germain et al. 2009; Shankar et al. 2014; van der Lee et al. 2017; Yuuki et al. 2023; Iravani et al. 2022; Yu et al. 2024; Agüera‐Ortiz et al. 2010; Hayashi et al. 2013; Wu et al. 2015; Hergert et al., 2021; Wang et al. 2016). These symptoms disrupt daily care routines and increase psychological stress for caregivers.
- Functional ability impairment: Impaired basic activities of daily living (BADL) (*n* = 4 studies) (Connors et al., 2019; Shankar et al. 2014; Yuuki et al., 2023; Chen et al,. 2011) and instrumental activities of daily living (IADL) (*n* = 3 studies) (Germain et al., 2009; Yuuki et al., 2023; Connors et al., 2019) increase caregiver burden due to caregivers’ physical workload and time investment.
- Cognitive and disease severity: severe cognitive decline (*n* = 6 studies) (D'Onofrio et al. 2015; Hergert et al., 2021; Kang et al. 2022; Yu et al. 2015; Chen et al. 2011; Germain et al. 2009) and longer disease duration (*n* = 2 studies) (Hayashi et al. 2013; Kang et al. 2022) lead to cumulative burden due to progressive loss of patient independence.

#### 3.3.2 Caregiver Factors

Female gender (*n* = 6 studies) and long care duration (*n* = 6 studies) are the most consistent risk factors, with mechanisms linked to role overload and emotional exhaustion.

- Socio‐demographic factors: Female caregivers (*n* = 6 studies) (D'Onofrio et al. 2015; Hergert et al. 2021; Iravani et al. 2022; Yuuki et al. 2023; Chen et al. 2011; Yu et al. 2024) bear heavier burdens due to greater emotional investment in care. Younger caregivers (*n* = 2 studies) (Germain et al. 2009; Shankar et al. 2014) and those with heavy financial burdens (*n* = 3 studies) (Chen et al. 2011; He et al. 2020; Xu et al. 2016) also face higher burdens.
- Psychological factors: Low symptom management self‐efficacy (*n* = 1 study) (Shankar et al. 2014) and depressive symptoms (*n* = 1 study) (Shankar et al. 2014) reduce caregivers’ coping ability, amplifying burden.
- Care‐related factors: Longer care duration, such as >8 h/d or 61 h/week (*n* = 6 studies) (D'Onofrio et al. 2015; Germain et al. 2009; Iravani et al. 2022; Yu et al. 2015; Chen et al. 2011; Xu et al. 2016), significantly increases burden, as does living with the patient (*n* = 1 study) (Wang et al. 2016).

#### 3.3.3 Environmental‐Social Factors

These factors act as buffers or amplifiers:

- Social support: One study (Yu et al. 2015) found that insufficient practical/emotional support reduces caregivers’ ability to cope with stress.
- Family functioning: Conflict or unclear role division (*n* = 1 study) (Yu et al. 2015) exacerbates burden by reducing mutual assistance among family members.

## 4 Discussion

### 4.1 Core Risk Factors and Mechanisms of Caregiver Burden

This scoping review, which encompassed 19 studies from at least 10 unique countries, confirms that caregiver burden in patients with cognitive dysfunction arises from multifactorial interactions, with patient neuropsychiatric symptoms and behaviors identified as a core risk factor across cultural contexts. This finding aligns with conclusions from global systematic reviews (Chiao et al. 2015; van den Kieboom et al. 2020), where 14 of the included studies (73.7%) verified that symptoms such as hallucinations, delusions, anxiety, and abnormal motor behaviors directly exacerbate caregiver burden. The underlying mechanism lies in the disruption of predictable care routines by these symptoms—for instance, nocturnal restlessness leads to caregiver sleep deprivation (Allegri et al. 2006), while delusions require additional emotional regulation and crisis intervention, thereby amplifying psychological stress and time investment. Notably, Allegri et al. reported a correlation coefficient of 0.482 (*p* < 0.001) between NPS and caregiver burden, highlighting the dominant role of NPS compared to other influencing factors (2006); however, the majority of studies identified a clear link between severe cognitive impairment and increased burden. A notable exception was the finding by Allegri et al. (2006), which reported no significant correlation. A critical appraisal of this discrepancy suggests a potential methodological explanation: the overwhelming impact of NPS may have confounded or masked the independent effects of pure cognitive test scores in multivariate analyses. This highlights a key limitation in some cross‐sectional studies: the challenge of disentangling the effects of highly correlated patient characteristics. It underscores that cognitive decline might not be a direct driver of burden per se, but rather that its effect is mediated or overshadowed by the more proximal and disruptive behavioral and psychological symptoms of dementia.

Patient functional dependence and caregivers' female gender are identified as key driving factors. Longitudinal studies (Connors et al., 2019; Germain et al., 2009) demonstrate that the progressive impairment of ADLs/IADLs increases the physical workload of caregiving, as caregivers gradually take over tasks such as feeding, dressing, and financial management. Among the included studies, 6 studies (31.6%) reported that female caregivers experience higher burden, a trend attributed to global gendered caregiving norms; women typically bear a disproportionate share of care responsibilities and exhibit greater emotional investment, leading to role overload and emotional exhaustion (van den Kieboom et al. 2020; Iravani et al. 2022). This is further supported by cross‐cultural data, which show that female caregivers in both Western (D'Onofrio et al. 2015) and Eastern (Chen et al. 2011) contexts experience higher levels of distress, indicating a universal gender‐based vulnerability.

Furthermore, environmental and social factors function as critical buffers or amplifiers of caregiver burden. Our synthesis identified that low social support and poor family functioning consistently emerged as significant environmental‐social risk factors. The mechanism through which low social support operates is one of resource depletion; the absence of practical assistance (e.g., respite care) and emotional backing isolates caregivers, leaving them without the necessary resources to cope with chronic stress (Yu et al. 2015). Similarly, poor family functioning, characterized by role conflict and a lack of clear responsibility division, exacerbates burden through intra‐familial strain. It reduces mutual assistance among potential family caregivers, thereby concentrating the care demands on a single individual and creating an environment of emotional discord (Wang et al. 2016). This underscores that caregiver burden is not merely an individual ordeal but is profoundly shaped by the broader social and familial ecosystem. The relative scarcity of studies (*n* = 2) quantifying these influences, compared to the wealth of literature on neuropsychiatric symptoms, highlights a critical evidence gap and a vital direction for future research.

### 4.2 Heterogeneity in Caregiver Burden: Cultural and Disease‐Specific Differences

#### 4.2.1 Cultural Heterogeneity

Cultural contexts significantly modulate the impact of certain risk factors, most notably caregiver education level. Western studies consistently link low educational attainment to higher caregiver burden, as limited access to caregiving resources and information reduces coping capacity (D'Onofrio et al. 2015; Shankar et al. 2014). In contrast, a Chinese study (Chen et al. 2011) found that highly educated caregivers face a greater burden, a phenomenon aligned with collectivist cultural values—educated individuals may internalize stronger filial responsibilities and hold higher expectations for care quality, resulting in ‘high expectation pressure’ (Liu et al. 2022). Another cultural difference manifests in social support systems: Western caregivers rely more on formal services, while Asian caregivers depend primarily on family support networks (Wang et al. 2016). Consequently, deficits in family function (e.g., role conflict) have a more pronounced impact on caregiver burden in Eastern contexts.

#### 4.2.2 Disease‐Specific Heterogeneity

Caregiver burden varies significantly across subtypes of cognitive dysfunction, following the hierarchy DLB > AD > VaD (Yuuki et al. 2023; D'Onofrio et al. 2015). DLB caregivers face the highest burden due to the combination of NPS and motor disorders, which create dual care demands (Yuuki et al. 2023). AD is characterized by progressive cognitive decline and persistent behavioral symptoms, while VaD has a more stable clinical course and fewer severe NPS, resulting in lower care intensity. This heterogeneity is supported by epidemiological data showing that DLB patients are associated with 1.5‐fold higher caregiver distress compared to AD patients (Zhang et al. 2022), emphasizing the need for disease‐specific interventions—for example, motor symptom management training for DLB caregivers and cognitive support strategies for AD caregivers.

### 4.3 Evaluation of Measurement Tools and Methodological Insights

ZBI and CBI are the dominant measurement tools, used in 84.2% of the included studies, due to their multidimensional assessment capabilities and cross‐cultural adaptability. The ZBI captures emotional, physical, and social dimensions of burden, while the CBI subdivides burden into time, developmental, physical, social, and emotional components, enabling precise identification of burden sources (Loo et al., 2022). Derivatives such as sZBI and J‐ZBI address practical needs, including simplified assessment and cultural adaptation (Kang et al. 2022; Yuuki et al. 2023), while complementary tools like NPI quantify caregiver distress specifically arising from patient NPS (van der Lee et al. 2017).

### 4.4 Implications for Practice, Policy, and Future Research

#### 4.4.1 Clinical Practice

Clinical interventions should target core risk factors to alleviate caregiver burden: (1) For patients, prioritize NPS management through behavioral therapy (Agüera‐Ortiz et al. 2010); (2) For caregivers, provide specialized skill training (e.g., de‐escalation techniques for managing patient agitation) and psychological support (e.g., cognitive‐behavioral therapy) to reduce emotional exhaustion (Gharavi et al. 2018); (3) Implement respite care services to alleviate the demands of 24/7 caregiving, particularly for caregivers living with patients (Wang et al. 2016).

#### 4.4.2 Policy Recommendations

Policy measures should address structural barriers to effective caregiving: (1) Provide financial subsidies for low‐income caregivers and cover care‐related expenses to reduce economic burden (He et al. 2020); (2) Establish community‐based care networks to share caregiving tasks and reduce the burden on individual caregivers (Wu et al. 2015); (3) Promote the development and adoption of intelligent assistive technologies (e.g., remote monitoring systems, care robots) to improve caregiving efficiency (Vollmer Dahlke and Ory 2020); (4) Adapt care services to cultural contexts, for example, recognizing the role of filial responsibilities in collectivist societies and offering family‐centered support programs.

#### 4.4.3 Future Research Directions

To address the gaps identified in this review, future research should prioritize the following directions: (1) conduct longitudinal and multicenter international studies, particularly in low‐ and middle‐income countries, to establish causal pathways and expand geographic representation; (2) validate and adapt measurement tools across diverse cultural contexts while developing standardized protocols to enhance cross‐study comparability; (3) expand research focus to include underrepresented populations such as male caregivers and caregivers of individuals with non‐Alzheimer's dementias; (4) develop and evaluate disease‐specific intervention models tailored to distinct cognitive impairment subtypes; and (5) explore the efficacy of digital health tools in supporting caregivers and reducing burden.

## 5 Study Limitations

While the studies included in this review provide a valuable foundational landscape of risk factors and identify consistent patterns across settings, several limitations must be considered as they affect the validity and generalizability of the findings.

### 5.1 Study Design and Search Limitations

The predominance of cross‐sectional studies limits causal inference between risk factors and caregiver burden. The scarcity of longitudinal designs restricts understanding of burden progression, and the absence of intervention trials prevents conclusions about mitigating identified risk factors. Furthermore, the restriction to six major databases and exclusion of grey literature may have resulted in publication bias and an incomplete evidence base.

### 5.2 Geographic Bias

The overrepresentation of high‐income countries and China, combined with English and Chinese language restrictions, limits the cross‐cultural validity of findings. This bias reduces generalizability to low‐ and middle‐income regions, where different healthcare systems and cultural norms may shape caregiver burden.

### 5.3 Measurement Issues

Inconsistent use of assessment tool versions and variations in scoring protocols reduced result comparability across studies. This heterogeneity may obscure the true effect sizes of risk factors and complicate data synthesis.

### 5.4 Population Gaps

Significant gaps exist in the evidence base, including underrepresentation of non‐Alzheimer's dementias and insufficient focus on male caregivers and caregivers of persons with early‐onset dementia. These gaps limit understanding of burden profiles in these specific populations.

## 6 Conclusion

This scoping review establishes that caregiver burden in cognitive dysfunction stems from interacting factors across three domains. Neuropsychiatric symptoms and behaviors (e.g., hallucinations, agitation) emerge as the most consistent core risk factors, while patient functional dependence and caregiver female gender constitute key drivers. These factors are compounded by prolonged care hours, financial strain, and limited social support. To address this complexity, future work should develop integrated intervention models combining personalized symptom management with caregiver skill‐building and respite services. These efforts must be informed by longitudinal studies to establish causal pathways and cross‐cultural comparisons to validate risk factors across diverse populations. Such an evidence‐based approach is essential for effectively reducing burden and advancing equitable care for cognitive dysfunction individuals globally.

## Author Contributions

**Liu Jinheng**: conceptualization, data curation. **Zhao Jingyi**: data curation, formal analysis. **Cui Shaomei**: formal analysis, writing – original draft. **Ye Danjuan**: writing – review and editing, visualization. **Wang Liansheng**: methodology, validation. **Chen Lixia**: conceptualization, methodology, supervision, writing – review and editing, project administration.

## Funding

The authors have nothing to report.

## Conflicts of Interest

The authors declare no conflicts of interest.

## Data Availability

This scoping review synthesized information from previously published research articles. All data supporting the results of this review are available within the cited literature.
